# Enterolignan Production in a Flaxseed Intervention Study in Postmenopausal US Women of African Ancestry and European Ancestry

**DOI:** 10.3390/nu13030919

**Published:** 2021-03-12

**Authors:** Susan E. McCann, Meredith A.J. Hullar, David L. Tritchler, Eduardo Cortes-Gomez, Song Yao, Warren Davis, Tracey O’Connor, Deborah Erwin, Lilian U. Thompson, Li Yan, Johanna W. Lampe

**Affiliations:** 1 Department of Cancer Prevention and Control, Roswell Park Comprehensive Cancer Center, Buffalo, NY 14263, USA; song.yao@roswellpark.org (S.Y.); warren.davis@roswellpark.org (W.D.); deborah.erwin@roswellpark.org (D.E.); 2 Cancer Prevention Program, Public Health Sciences Division, Fred Hutchinson Cancer Research Center, Seattle, WA 98109, USA; mhullar@fredhutch.org (M.A.J.H.); jlampe@fredhutch.org (J.W.L.); 3 Department of Biostatistics, University at Buffalo, Buffalo, NY 14214, USA; davidtritchler@gmail.com; 4 Department of Biostatistics and Bioinformatics, Roswell Park Comprehensive Cancer Center, Buffalo, NY 14263, USA; Eduardo.cortesgomez@roswellpark.org (E.C.-G.); li.yan@roswellpark.org (L.Y.); 5 Department of Medicine, Roswell Park Comprehensive Cancer Center, Buffalo, NY 14263, USA; tracey.oconnor@roswellpark.org; 6 Department of Nutritional Sciences, University of Toronto, Toronto, ON M5S 1A8, Canada; lilian.thompson@utoronto.ca

**Keywords:** dietary intervention, women, ethnicity, microbiota, enterolignans

## Abstract

Lignans are phytochemicals studied extensively as dietary factors in chronic disease etiology. Our goal was to examine associations between the gut microbiota and lignan metabolism and whether these associations differ by ethnicity. We conducted a flaxseed (FS) dietary intervention in 252 healthy, postmenopausal women of African ancestry (AA) and European ancestry (EA). Participants consumed ~10 g/d ground flaxseed for 6 weeks and provided overnight urine collections and fecal samples before and after intervention. The gut microbiota was characterized using 16S rRNA gene sequencing and differences in microbial community composition compared by ethnicity and intervention status. We observed a significant difference in the composition of the microbiota measured as beta diversity (*p* < 0.05) between AA and EA at baseline that was attenuated with FS consumption. Genera that were significantly associated with ENL production (e.g., *Klebsiella*, *Lactobacillus*, *Slackia*, *Senegalimassilia*) were unique to each group. Bacteria (e.g., *Fusobacteria*, *Pyramidobacter* and *Odoribacter*) previously associated with colorectal cancer and cardiovascular disease, both diet-related chronic diseases, were unique to either AA or EA and were significantly reduced in the FS intervention. This study suggests that ethnic variation in ENL metabolism may be linked to gut microbiota composition, and its impact on disease risk deserves future investigation.

## 1. Introduction

Lignans are polyphenolic compounds ubiquitous in plant foods, with the highest concentrations found in seeds (particularly flaxseed [FS]), whole grains, vegetables and fruits [1,2], and have been studied as dietary factors in cancer etiology given structural and functional similarities to endogenous estrogens and anti-carcinogenic activities [3]. Plant lignans are metabolized to the physiologically active enterolignans by bacteria in the mammalian gut [4,5,6,7,8,9]. For example, the predominant lignan in FS is secoisolariciresinol diglucoside (SDG) which is deglycosylated to secoisolariciresinol (SECO) by bacterial genera such as *Bacteroides* and *Klebsiella* [10,11,12]. After hydrolysis, the aglycones can be absorbed, or dehydroxylated or demethylated into enterodiol (ED) or enterolactone (ENL) by a variety of bacteria, including *Blautia producta, Eubacterium callanderi, Eubacterium limosum,* members of the family Coriobacteriaceae, *Bacteroides methylotrophicum*, or certain strains of Lactobacillus [10,12]. ED can be further metabolized to ENL, which is the predominant circulating enterolignan [13]. Efficiency of conversion of ED to ENL varies by individual [14] and may be, in part, associated with specific composition of the gut microbiota. While the bacteria above have been associated with ENL production, recent studies suggest that these metabolic reactions can be carried out by many different bacteria [13]. In fact, bacterial production of enterolignans, in general, demonstrates a substantial amount of variation between individuals, some of which may be related to specific gut microbial community composition (GMC) as well as other participant characteristics such as age, diet, and health status [5,14,15,16,17,18].

Given the contribution of individual characteristics to variation in lignan metabolism, ethnicity may also be an important effect modifier, as ethnicity can incorporate genetic diversity, microbial diversity, and sociocultural and lifestyle factors, including diet. As mentioned above, as lignans have structural and functional similarities to endogenous estrogens as well as anticarcinogenic activities, determination of influences on lignan production are relevant to postmenopausal women who are at higher risk for hormone-related cancers. Therefore, we conducted a FS dietary intervention study in healthy postmenopausal women to determine how variation in GMC affects the production of the bacterially derived enterolignans, ENL and ED, and how these associations differ for non-Hispanic US women of African ancestry (AA) and European ancestry (EA).

## 2. Materials and Methods

The study design and methodology were described in detail elsewhere [15]. Briefly, we conducted a randomized, crossover FS intervention study in 138 EA and 120 AA, healthy, postmenopausal women, ages 45–75 years, recruited from the western New York region with no history of use of antibiotics, hormone replacement therapy, non-prescription hormones or herbal supplements for menopausal symptoms, or flaxseed supplements in the prior 2 months. Participants were randomized to either: group (1) 10 g/d of ground FS for 6 weeks (*n* = 93; 52 EA, 41 AA), or group (2) maintenance of usual diet (*n* = 96; 61 EA, 35 AA), and after a 2-month washout crossed over to the other condition for an additional 6 weeks. Overnight urine collections and fecal samples were collected at the beginning and end of each 6-week intervention period, for a total of 4 collections per participant over the 5-month study period. Overnight urine collections were obtained for determination of SECO, ENL, and ED. Fecal samples were self-collected at home using the Commode Specimen Collection system (Fisher Scientific, Waltham, MA, USA) and a feces collection tube with scoop in the lid (Sarstedt, Newton, NC, USA), glass beads (3 mm; Fisher Scientific) and 5 ml RNAlater (Lifesciences Technologies, Leawood, KS, USA) for preservation of microbial nucleic acids. All biospecimens were frozen at −80 °C in the DataBank and BioRepository (DBBR) laboratories at Roswell Park Comprehensive Cancer Center (Roswell Park; Buffalo, NY, USA) until assay. This study was registered at clinicaltrials.gov as NCT01698294. The study protocol was conducted according to established ethical guidelines, approved by the Roswell Park Institutional Review Board, and women provided signed, informed consent before participation.

Accrual and retention goals were 100 each EA and AA completing all study activities. Participants self-identified as either non-Hispanic African American or White. Ancestry informative markers confirmed that participants were genetically homogenous with self-reported ancestry (hereafter referred to as ‘ethnicity’) [15]. Of 933 women screened for eligibility, 312 (33%) women were not eligible, 220 were eligible but did not participate (35% of eligible women), 292 (47% of eligible women) consented, and 258 (88% of consenting women) were randomized (120 AA, 138 EA). Retention was greater among EA women: 76 (63%) AA and 113 (82%) EA women completed all 4 study visits. Primary reasons for not completing the study included starting antibiotics or other medical reason (40.2%), or passive refusal wherein the participant did not return after the baseline (enrollment) visit (17.7%).

### 2.1. Intervention

Ground whole FS was provided as a single lot from a single source (Heartland Flax, Valley City, ND, USA; 32.62 mg/g SDG and 0.26 g/g total dietary fiber). Heartland Flax stored the whole FS and ground (milled through a 14 mesh US Standard sieve screen) and packaged it for the duration of the study. Participants were provided with one 500 g bag of ground FS and standardized scoops with instructions to consume one scoop per day (~10 g/d) mixed into water or juice. Any unused FS was returned to the study coordinator at the end of the 6-week period to monitor adherence. During the 5-month study period, women were instructed to avoid other dietary sources of FS such as breads and cereals with flaxseed added. In addition to returning non-consumed FS, adherence was assessed through determination of urinary lignan levels. Compliance was good with 91% (AA) and 95% (EA) of planned FS doses consumed.

Study participants completed interviewer-administered questionnaires including basic demographic information, medical and reproductive histories, medication and dietary supplement use, family history of chronic disease, physical activity, cigarette smoking history, and other epidemiologic data relevant to diet and cancer. BMI (kg/m^2^) and body composition (lean and fat mass) were assessed at each visit by a bioimpedance analysis (BIA) system (50 kHz BC-418, Tanita Corporation, Tokyo, Japan). Dietary data were collected via 12 telephone-administered 24-hour dietary recalls administered randomly throughout the 5-month active intervention period and analyzed with the University of Minnesota Nutrient Data Systems for Research (NDSR) dietary analysis program. 

### 2.2. Outcomes

Urinary SECO, ED, and ENL were assayed by isotope dilution gas chromatography-mass spectrometry in the SIM mode (HP 6890 GC, HP 5973 MSD; Agilent Technologies, Palo Alto, CA, USA) as described previously [15]. All lignan measures were normalized to urinary creatinine levels to adjust for urine concentration. Stool samples were thawed, homogenized, and genomic DNA was extracted as described previously [19]. DNA concentrations and purity were determined using the NanoDrop 8000 Spectrophotometer (ThermoFisher Scientific) and gel electrophoresis. Working stocks were diluted in AE buffer (QIAGEN, Germantown, MD, USA) from genomic DNA and samples were stored at −20 °C until shipped for sequencing. Pooled in-lab designed quality control samples were included in triplicate to assess variation in library preparation and sequencing batches [20]. The V1–V3 region of the 16S rRNA gene was sequenced using the Illumina MiSeq platform to obtain 2 × 300 bp paired-end reads. Fecal bacterial DNA extraction did not meet quality control standards for 15 participants and 5 participants were excluded for higher baseline vs. post-intervention enterolignan excretion (post-preintervention difference ≤−10 nmol/mg creatinine). Therefore, the final sample sizes for the present analyses were 228 completing baseline assessments and 170 completing all 4 visits.

## 3. Bioinformatic and Statistical Analysis

To assign bacterial taxonomy, sequences were processed using QIIME v.1.8 [21] and classified [22,23] using the SILVA (1.32) 16S rRNA gene reference database [24,25,26]. Bioinformatic processing of 16S rRNA gene sequences resulted in a total of 31,633,561 sequences (24% were below QC parameters). The average length was 507 bp ± 51 and the average number of sequences per sample was 28,387 ± 17,555. Alpha diversity measures Shannon diversity index [27] and beta diversity matrices (unweighted and weighted UniFrac) [28,29] were calculated in QIIME after rarefying to 8000 sequences per sample. All data matrices were exported for statistical analysis.

16S sequence data were first summarized to operational taxonomic units (OTUs) at the species level (97% similarity). We identified 10 phyla, 115 genera and 689 OTUs from 16S rRNA gene sequences in the samples. Shannon diversity index was estimated for alpha diversity scores using phyloseq package (v1.28.0) [30]. For beta diversity, Bray-Curtis dissimilarity score, weighted and unweighted UniFrac [28,29] were visualized with non-metric multidimensional scaling and were estimated and tested using the PERMANOVA procedure (5000 permutations) implemented by the vegan package (v2.5.6; https://cran.ism.ac.jp/web/packages/vegan/vegan.pdf). Additional summary statistics, tests, and visualizations for alpha and beta diversity were performed by ethnicity and intervention status. Statistical analyses and comparisons to detect differential abundance of genera were carried out using pscl package (v1.5.2; ftp://ftp.sam.math.ethz.ch/sfs/R-CRAN/web/packages/pscl/pscl.pdf). This methodology implements a likelihood ratio test using a hurdle generalized linear model (hurdle GLM) assuming the outcome variable, in this case genera, has a negative-binomial distribution. The choice of using a hurdle model was based on the sparseness of the genera counts (Supplemental Figure S1). Differential genera abundance was carried out between the different groups of interest (e.g., intervention status, ethnicity) accounting for baseline age (y), BMI (kg/m^2^), total dietary fiber (g/d), energy intake (kcal/d), and total FS consumption during the intervention period (g/intervention period). Post-intervention models were further adjusted for baseline ENL excretion. Genera with <1% zeros across samples were fit with a negative-binomial generalized linear model (GLM); otherwise, a hurdle model was used. Genera with >85% zeros across samples were considered low quality and removed. From 115 genera, 8 and 6 were removed when fitting AA and EA models, respectively. Forest plots were used to visualize important genera (FDR < 0.05) which were then color-coded for phylum and displayed by ethnicity. Statistical analyses were performed in R (3.6.1) and related programs specific to running the programs listed above. 

We used ElenMatchR (https://github.com/jbisanz/ElenMatchR) [31], a random forest classifier, to identify in silico whether genes involved in lignan metabolism were present in the reference genomes of related members of the Family Coriobacteriaceae associated with urinary ENL in our data. We were particularly interested in the two-gene loci benzyl-ether reductase (*ber*) and its putative transcriptional regulator responsible for ENL production from lignans. We analyzed the genomes for two *Senegalimassilia* species, nine *Slackia* species, and two *Collinsella* species. We set parameters for a clustering threshold of 30% amino acid identity and 50% coverage (Supplemental Table S1).

Demographic and personal characteristics by ethnicity were described using basic descriptive statistics. Differences in enterolignan excretion by randomization group across visits were assessed with repeated measures mixed models adjusting for age, BMI, and amount of flax consumed during intervention and stratified by ethnicity and randomization group.

## 4. Results

We previously observed no statistically significant differences in age, BMI, cigarette smoking status, education, or lignan excretion between women who completed the study vs. women with baseline data only [15]. As shown in Table 1, compared to EA women, AA women had higher BMI, higher prevalence of current cigarette smoking, less education, lower levels of ENL and SECO, and lower daily intakes of total energy, dietary fiber, whole grains, and vegetables (all *p* < 0.05).

**Figure 1:**
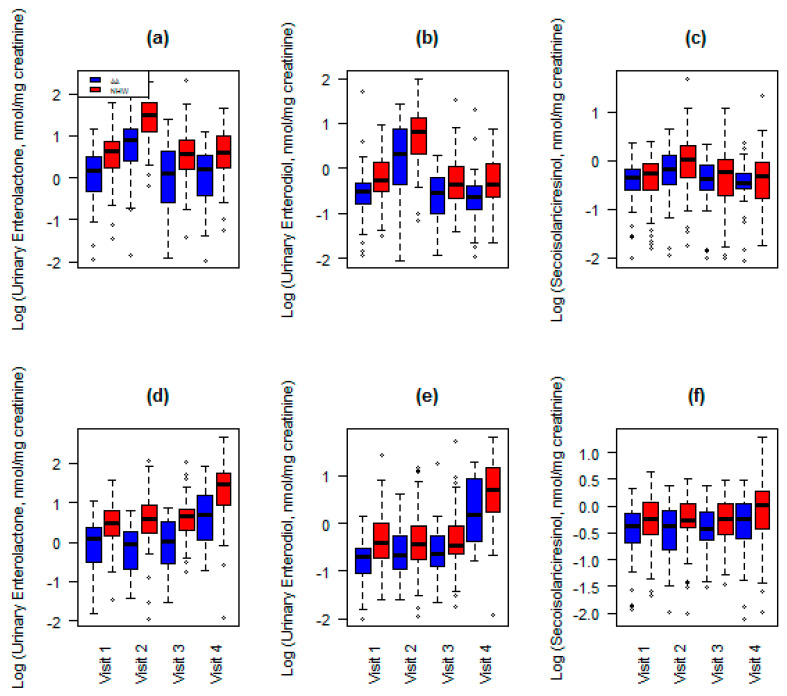
Urinary lignan excretion by randomization group, visit, and ethnicity compared with repeated measures ANOVA adjusting for age, body mass index, and flaxseed (FS) consumed. Blue bars represent women of African ancestry; red bars represent women of European ancestry. Figure 1a–c represent enterolactone (ENL) (**a**), enterodiol (ED) (**b**), and secoisolariciresinol (SECO) (**c**) excretion, by visit, for women assigned to treatment group 1 (FS first 6 weeks). As expected, excretion of these lignans increases between visits 1 and 2 and is relatively unchanged between visits 3 and 4. Figure 1d–F represent ENL (**d**), ED (**e**), and SECO (**f**) excretion, by visit, for women assigned to treatment group 2 (FS last 6 weeks). Similar to effects observed in group 1, excretion of these lignans increased between visits 3 and 4 in response to FS supplementation. ENL, ED and SECO excretion increased statistically significantly after FS in both groups (*p* < 0.0001).

Urinary lignan excretion by ethnicity, randomization group, and visit are shown in Figure 1. Figure 1a–c represent participants in group 1 who received FS during the first 6 weeks of the study. As expected, excretion increased between visits 1 and 2 in response to FS consumption for ENL (Figure 1a; *p* < 0.001, AA and EA), ED (Figure 1b; *p* < 0.001, AA and EA), and SECO (Figure 1c; *p* = 0.0069 and *p* < 0.0001, AA and EA, respectively). Figure 1d–f represent participants in group 2 who received FS during the last 6 weeks of the study. Similar to the effects observed in group 1, excretion increased between visits 3 and 4 in response to FS consumption for ENL (Figure 1d; *p* < 0.001, AA and EA), ED (Figure 1e; *p* < 0.001, AA and EA), and SECO (Figure 1f; *p* = 0.17 and *p* = 0.0048, AA and EA, respectively). Interestingly, at each time point, excretion was higher among EA compared to AA: ENL (*p* < 0.0001, group 1 and 2), ED (*p* < 0.001, group 1 and 2) and SECO (*p* < 0.001 and *p* = 0.0024, group 1 and 2, respectively).

**Figure 2:**
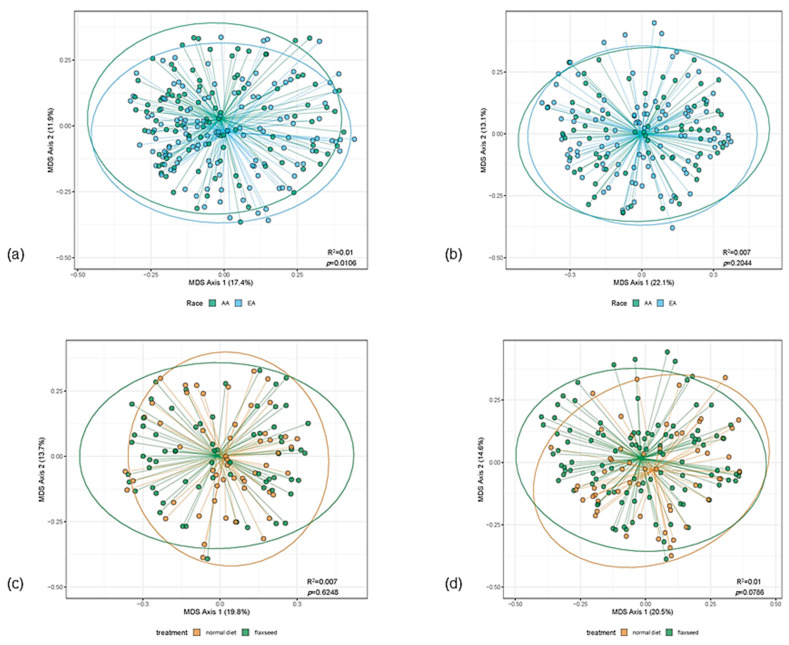
Beta diversity by ethnicity stratified by intervention status and by intervention status stratified by ethnicity. (**a**) shows beta diversity by ethnicity at baseline; (**b**) shows beta diversity by ethnicity after flaxseed (FS) intervention; (**c**) shows beta diversity by intervention status among women of African ancestry (AA); (**d**) shows beta diversity by intervention status among women of European ancestry (EA). Community structure differs by ethnicity at baseline (Figure 2a; *p* = 0.0106), but not after FS intervention (Figure 2b; *p* = 0.2044). A small amount of variation in community structure is explained by ethnicity differences (Figure 2a, R^2^ = 0.01 and Figure 2b, R^2^ = 0.007). Community structure does not differ significantly by intervention status for AA (Figure 2c) or EA (Figure 2d) women.

Alpha diversity (Shannon Diversity Index) did not differ between AA and EA women, nor from baseline to post-intervention (data not shown). As shown in Figure 2, beta diversity, measured using UniFrac or the Bray-Curtis distant metric, differed by ethnicity at baseline (Figure 2a, *p* = 0.019), but not after FS consumption (Figure 2b). Although statistically significantly different at baseline, only a small amount of variation in community structure was explained by ethnicity in this study (R^2^ = 0.01). Community structure did not differ by intervention status for AA or EA women (Figure 2c,d), nor did overall community structure vary with ENL excretion (data not shown).

**Figure 3:**
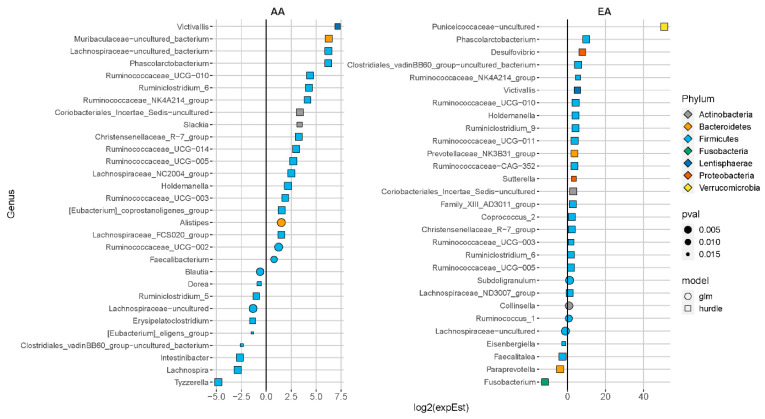
Differentially abundant genera associated with enterolactone excretion at baseline in women of African ancestry (AA) and European ancestry (EA); adj *p* < 0.05. Genera with less than 1% zeros across samples were fit with a negative-binomial generalized linear model (GLM) otherwise, a hurdle model was used. Models were adjusted for baseline age, body mass index (kg/m^2^), total dietary fiber (g/d), energy intake (kcal/d), and total flaxseed consumption during the intervention period (g).

**Figure 4:**
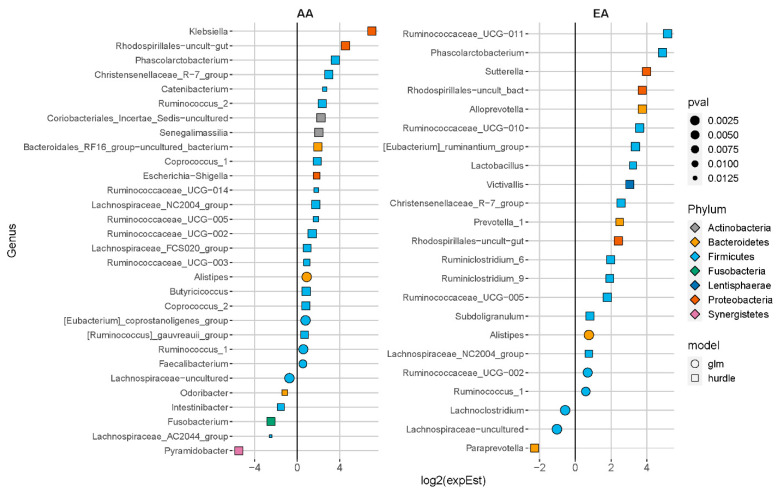
Differentially abundant genera associated with enterolactone (ENL) excretion after flaxseed (FS) intervention; padj < 0.05. Genera with less than 1% zeros across samples were fit with a negative-binomial generalized linear model (GLM) otherwise, a hurdle model was used. Models were adjusted for baseline age, body mass index (kg/m^2^), total dietary fiber (g/d), energy intake (kcal/d), total FS consumption during the intervention period (g), and baseline ENL excretion.

At baseline, 12 genera associated with ENL excretion were found in both AA and EA. In contrast, after FS intervention nine bacteria were found in common in AA and EA participants who excreted ENL (Supplemental Figure S2). The differentially abundant genera associated with ENL excretion by ethnicity are shown in Figure 3 (baseline) and Figure 4 (after FS). Genera positively associated with ENL regardless of ethnicity included genera in Christensenellaceae, Prevotellaceae, Ruminococcaceae, and some Lachnospiraceae (adj. *p* < 0.05). At baseline in AA women, *Alistipes* (adj. *p* = 0.0003)*,* a Lachnospiraceae genera (adj. *p* = 0.000008), and *Slackia* (adj. *p* = 0.04) were enriched and *Lachnospira* (adj. *p* < 0.0001) and *Tyzzerella* (adj. *p* < 0.0001) were reduced. *Fusobacterium* (adj. *p* = 0.01) and *Paraprevotella* (adj. *p* = 0.004) were reduced and a Puniceicoccaceae genera (adj. *p* < 0.0001) was enriched in EA at baseline. In the AA women after FS, *Klebsiella* (adj. *p* < 0.0001) was enriched in participants who excreted ENL whereas *Fusobacteria* (adj. *p* = 0.009), *Odoribacteria* (adj. *p* = 0.035), and *Pyramidobacter* (adj. *p* = 0.007) were reduced. In contrast, *Lactobacillus* (adj. *p* = 0.02), two *Ruminiclostridium* genera (adj. *p* < 0.01) and *Victivalis* (adj. *p* = 0.001) were enriched and *Lachnoclostridium* (adj. *p* = 0.005) was inversely associated with ENL in EA after FS. 

We found that in the AA women, *Slackia* (adj. *p*-value = 0.04) was enriched at baseline and *Senegalimassilia* (adj. *p* = 0.0005) was enriched after the FS intervention (Figure 3 and Figure 4, respectively). Reference genomes of closely related taxa in the Coriobacteriaceae family support that both genera contain genes associated with ENL production (Supplemental Table S1). In contrast, in the EA women, the genera *Collinsella* (adj. *p* = 0.01) was enriched in the baseline samples, although not after the FS intervention (Figure 3 and Figure 4, respectively). Furthermore, the reference genomes of the different species of the genera *Collinsella*, did not contain genes associated with ENL production.

## 5. Discussion

In this FS intervention study conducted in healthy postmenopausal women, we observed statistically significant differences in the amount of ENL produced in EA and AA women both before and after the intervention. Despite few statistically significant differences in alpha and beta diversity measures, several genera associated with ENL excretion differed by ethnicity at both timepoints. At baseline, 18 genera among AA participants and 17 genera among EA participants were significantly differentially abundant; 12 of these were in common between the two groups. However, after FS intervention, 21 genera among AA and 14 genera among EA participants were significantly differentially abundant with only 9 genera in common. These findings suggest that, at least for postmenopausal women, ethnicity-specific microbial community composition may be important in the differences observed in ENL excretion between AA and EA. 

Others have reported higher enterolignan excretion in non-Hispanic whites compared to non-Hispanic blacks [32]. Dietary lignan intake may be affected by cultural and ethnic differences in diet patterns [33], and cross-sectional studies support that the types and amounts of lignans ingested influence production of ENL [32,34,35,36,37,38,39]. The impact of healthier food choices on ENL was confirmed independently by Kilkkinen et al. who showed positive associations between the intake of whole grains, fruit and vegetables, and serum ENL concentrations [40,41]. Although in our study dietary fiber, fruit and vegetable intakes were significantly lower in the AA women compared to EA women, adjustment for these variables had little impact on our findings. In contrast, Valentin-Balsini et al. found no difference in ENL excretion associated with ethnicity, although sociodemographic and lifestyle factors (poverty income ratio (PIR), BMI, and physical activity) were important [32]. Viewed in combination with the findings of Kerver et al. (43), who reported that in U.S. adults (NHANES III), PIR was positively correlated with higher scores on a dietary pattern typified by higher intakes of whole grains, fruit, and vegetables, sociodemographic factors likely contribute to food choices and influence ENL production.

Given that ENL production is also affected by microbial conversion efficiency of different lignans to enterolignans [42,43,44,45], the gut microbiota may contribute to inter-individual and population-specific variation in enterolignan exposures. Gut microbial diversity may be responsive to the variety of dietary components resulting from cultural and ethnic differences in dietary patterns [33], although in our study, alpha diversity did not differ in the AA or EA at baseline or in response to FS. However, beta diversity at baseline did differ between EA and AA, albeit with a small effect size which was attenuated after the intervention. Our findings are consistent with another intervention study reporting no difference in alpha or beta diversity after lignan supplementation [18] but are in contrast to a cross-sectional study reporting an association between microbial diversity and ENL production in participants maintaining habitual diet [46]. 

Inter-individual variation in ENL production by bacterial metabolism may be a result of different patterns of microbial composition, wherein either a single genus or a consortium of multiple genera can metabolize plant lignans to ENL. [5,6,13,47]. Our data support the findings of other studies reporting that inter-individual variation and population-specific difference in ENL production is linked to variation in microbiome composition [13,47]. In the AA women, *Klebsiella* was significantly enriched with the FS intervention (Figure 4). This facultative anaerobe is historically associated with fatal nosocomial infections, but recent studies show that non-pathogenic strains (*Klesbisella* sp. Strain S1) convert SDG to SECO [12]. In the EA women, *Lactobacillus* was significantly enriched after FS. Gram-positive bacteria including *Lactobacillus* have been associated with deconjugation of lignans and three strains of *Lactobacillus* were shown to produce ED and ENL from flax extracts [13,47]. Moreover, some *Lactobacillus* strains have strong substrate specificity for SECO [42,47]. Coriobactericeae, enriched after FS in AA but not in EA, has genera associated with consortia that degrade another plant lignan, matairesinol, and share some of the intermediate conversion steps, such as benzyl ether reduction, in the production of ENL from SDG [13] which was supported in our bioinformatic analysis. ENL was positively associated with *Ruminococcus* groups in both EA and AA, although different genera were enriched in each group. *Ruminococcus* are an integral part of consortia involved in the anaerobic fermentation of phytochemicals including conversion of ED to ENL [48]. 

Members of Family Coriobacteriaceae are part of consortia that metabolize a variety of complex phytochemicals, including lignans [13]. We found several Coriobactericeae members associated with ENL (Figure 4), the distribution of which varied by ethnicity. In AA, *Slackia* was associated with ENL at baseline and *Senegalimassilia* after FS. The reference genomes for both genera contain genes associated with ENL production (Supplemental Table S1), specifically the two-gene loci benzyl-ether reductase (*ber*) and its putative transcriptional regulator responsible for ENL production from a lignan [13]. At baseline, both *Collinsella* and an uncultured genus in Coriobacteriaceae were enriched in EA women. In contrast, our bioinformatic analysis suggested that species related to *Collinsella* did not contain genes involved in ENL production in the EA although they are members of the family Coriobacteriaceae (Supplemental Table S1). Furthermore, after FS, none of the Coriobacteriaceae were enriched in the microbiome of EA participants. The 16S rRNA gene patterns and our in silico genomic analysis suggest specialization within Coriobacteriaceae that may explain some of the ethnic differences in ENL production, although we are the first to our knowledge to show differences in abundance of these genera associated with ethnicity. Others have found strain-specific variation in carriage of genes involved in ENL production in another Coriobacteria, *Eggerthella lenta* [13]. These observations warrant further investigation using metagenomic and metatranscriptomic approaches to identify inter-individual and population-specific differences in strains, specific genes, and gene expression associated with ENL production that may impact host health. 

A diet high in fiber, whole grains and vegetables impacts microbial saccharolytic fermentation and metabolism of phytochemicals to short chain fatty acids (SCFA) and other metabolites. Both EA and AA showed enrichment in genera in Christensenellaceae, Prevotelleaceae, Ruminococcaeceae, and some Lachnospiraceae [49,50], which are involved in SCFA production [50]. Christensenellaceae, a highly heritable microbe [51], was enriched after FS in both AA and EA. Large-scale diet studies have associated Christensenellaceae with healthy dietary habits [52,53,54] and in human dietary interventions involving prebiotic fibers [55,56,57]. Members of the Christensenellaceae family ferment sugars in the gut to SCFA and other fermentation products such as H_2_ and CO_2_ [58]. In our study, in both AA and EA, potential fermentation of fiber to succinate was supported by enrichment in members of Bacteroides and Prevotellaceae that produce succinate, and subsequent metabolism of succinate to propionate by *Alistipes* and *Phaseolarticum*. Metabolism to succinate and propionate has been associated with improved glucose homeostasis. [59,60] Lachnospiraceae NC2004 [61], a genus associated with ring cleavage of polyphenolic compounds found in high-fiber foods, was also enriched after FS. Additionally, within EA, *Victivallus*, a cellobiose degrading bacteria, and *Ruminiclostridia*, a lignocellulose-degrading bacteria, were enriched after FS and may reflect higher dietary fiber, whole grains, and vegetable intake by EA in our study (Table 1). 

Our findings suggest that differential metabolism of lignans in AA and EA women has implications for disparities in the prevalence of diet-related chronic diseases. *Pyramidobobacter*, *Odoribacter* and *Fuosbacteria,* associated with genotoxic sulfide production and CRC, [62] were reduced in AA after FS. In contrast, *Lachnoclostridium*, a genus associated with secondary bile acid metabolism, was inversely associated with ENL production in EA. Although many of the bacteria in common between AA and EA after FS supplementation are associated with saccharolytic fermentation and phytochemical metabolism, some of the genera unique to each race are associated with diet-related chronic diseases. Our data suggest that observed differences in ENL production and microbiome composition between AA and EA could be relevant to population-specific differences in chronic disease prevalence. For example, cross-sectional studies have reported an inverse relationship between ENL excretion and circulating secondary bile acids [63,64], bacterial metabolites associated with increased risk of CVD, cirrhosis [65], and CRC [45,64]. In our study, *Lachnoclostridium* was inversely associated with ENL production in EA. *Lachnoclostridium* includes species involved in the 7α-dehydroxylation of cholic acid to the secondary bile acid dehydroxycholic acid (DCA) [66], and a recent metagenomic analysis demonstrated that ENL-producing microbial consortia also expressed genes involved in secondary bile acid metabolism [63]. Further exploration is needed to investigate whether a diet high in lignans may alter the microbiome sufficiently to reduce the exposure to the amount and type of secondary bile acids linked to disease risk.

## 6. Conclusions

We present herein one of the few studies to examine differences in ENL exposure in a dietary intervention with FS and associations with the gut microbiome that vary by ethnicity. To our knowledge, our FS intervention study is the largest to date that includes similar numbers of AA and EA women, thus providing important insight into potential population-specific differences in the impact of dietary components on microbial-host metabolism. However, since the study was limited to postmenopausal women, we do not know if our results are applicable to AA and EA men. Finally, using taxon-based methodology, we were unable to directly identify species or strain differences and genome content linked to functional gene pathways involved in ENL production. Future analyses should include both sexes as well as functional analysis of species and strains in the intervention response.

## Figures and Tables

**Table 1:** Baseline descriptive characteristics of women participating in a flaxseed intervention study ^1^.

	Overall (*n* = 170)	AA (*n* = 67)	EA (*n* = 103)
	Mean ± SD		
Age, y	59.8 ± 6.1	59.5 ± 6.1	60.0 ± 6.2
BMI, kg/m^2^	29.9 ± 7.6	33.1 ± 8.1 ^2^	27.9 ± 6.5
Enterolactone, nmol/mg Cr	4.3 ± 6.5	2.2 ± 2.6 ^2^	5.6 ± 7.8
Enterodiol, nmol/mg Cr	0.7 ± 1.1	0.4 ± 0.4 ^2^	1.0 ± 1.3
Secoisolariciresinol, nmol/mg Cr	0.6 ± 0.6	0.5 ± 0.5 ^3^	0.8 ± 0.7
Energy, kcal/d	1628 ± 386	1552 ± 359 ^3^	1678 ± 396
Dietary fiber, g/d	17.6 ± 6.7	15.0 ± 5.9 ^2^	19.3 ± 6.7
Whole grains, g/d	1.0 ± 0.7	0.8 ± 0.6 ^3^	1.1 ± 0.8
Vegetables, g/d	3.1 ± 1.5	2.6 ± 1.2 ^2^	3.3 ± 1.6
Fat, g/d	66.5 ± 18.4	64.4 ± 18.5	67.9 ± 18.4
Protein, g/d	67.3 ± 17.0	62.8 ± 17.2 ^3^	70.1 ± 16.4
	*n* (%)		
BMI Category (kg/m^2^)			
<25	44 (25.9)	8 (11.9) ^2^	36 (35.0)
25–29.9	46 (27.1)	13 (19.4)	33 (32.0)
30–34.9	42 (24.7)	24 (35.8)	18 (16.8)
35–39.9	21 (12.4)	12 (17.7)	10 (9.4)
≥40	17 (10.0)	10 (14.7)	7 (6.5)
Smoking status			
Never smoker	79 (46.5)	25 (36.8) ^2^	56 (52.3)
Former smoker	60 (35.3)	19 (27.9)	43 (40.2)
Current smoker	31 (18.2)	24 (35.3)	8 (7.5)
Education			
≤High school	31 (18.2)	17 (25.0) ^2^	15 (14.0)
Vocational/technical/some college/associates	60 (35.3)	35 (51.5)	28 (26.2)
Bachelors degree	38 (22.4)	9 (13.2)	30 (28.0)
Graduate degree	41 (24.1)	7 (10.3)	34 (31.8)

AA African ancestry, EA European ancestry, Cr creatinine, y year, BMI body mass index; ^1^ participants with 16S rRNA gene data, completing all study visits; ^2^ *p* < 0.0001; ^3^ *p* < 0.05; differences by ethnicity calculated with χ^2^ for categorical and t-test for continuous variables.

## Data Availability

Data described in the manuscript, code book, and analytic code will be made available upon request pending application and approval.

## Supplementary Materials

The following are available online at https://www.mdpi.com/2072-6643/13/3/919/s1, Figure S1: Proportion of zeroes per sample (Figure 1a) and per OTU (Figure 1b), Figure S2: Venn diagram representing numbers of genera statistically significantly associated with ENL at baseline (Figure S2a) and after intervention (Figure S2b) by ethnicity, Table S1: Discovery of genetic determinants in the metabolism of lignans in genera associated with urinary ENL in our dietary intervention. Genome accession number for species related to genera associated with ENL in this study and presence or absence (+/−) of the benzyl ether reductase (*ber*) and putative transcription factor identified using the random forest classifier ElenMatchR (https://doi.org/10.1016/j.chom.202E0.04.006).

## Abbreviations

AA	African ancestry
BMI	body mass index, kg/m^2^
CRC	colorectal cancer
CVD	cardiovascular disease
EA	European ancestry
GMC	gut microbial community
FDR	false detection rate
FS	flaxseed
ER	estrogen receptor
OTU	operational taxonomic unit
SECO	secoisolariciresinol
SDG	secoisolariciresinol diglucoside
ED	enterodiol
ENL	enterolactone
