# Supplementary material for: Enterolignan Production in a Flaxseed Intervention Study in Postmenopausal US Women of African Ancestry and European Ancestry
Source: Nutrients. 2021 Mar 12;13(3):919. doi: 10.3390/nu13030919 (PMC8001909; doi:10.3390/nu13030919)
Supplement: Supplementary file 1 [file nutrients-13-00919-s001.pdf]

Supplemental data

Table S1. Discovery of genetic determinants in the metabolism of lignans in genera associated with urinary ENL in our dietary intervention. Genome accession number for species related to genera associated with ENL in this study. and presence or absence (+/-) of the benzyl ether reductase (*ber*) and putative transcription factor identified using the random forest classifier ElenMatchR (https://doi.org/10.1016/j.chom.202E0.04.006).

Genome accession number	+/-
Collinsella aerofaciens ATCC 25986	-
Collinsella aerofaciens CaUC5	-
Senegalimassilia anaerobia AP69FAA	+
Senegalimassilia anaerobia JC110	+
Slackia equolifaciens DSM 24851	+
Slackia exigua DSM 15923	+
Slackia exigua UCSF 15923	+
Slackia faecicanis DSM 17537	+
Slackia heliotrinireducens DSM 20476	+
Slackia heliotrinireducens UCSF 20476	+
Slackia isoflavoniconvertens DSM 22006	+
Slackia isoflavoniconvertens OB21 GAM31	+
Slackia piriformis DSM 22477	+

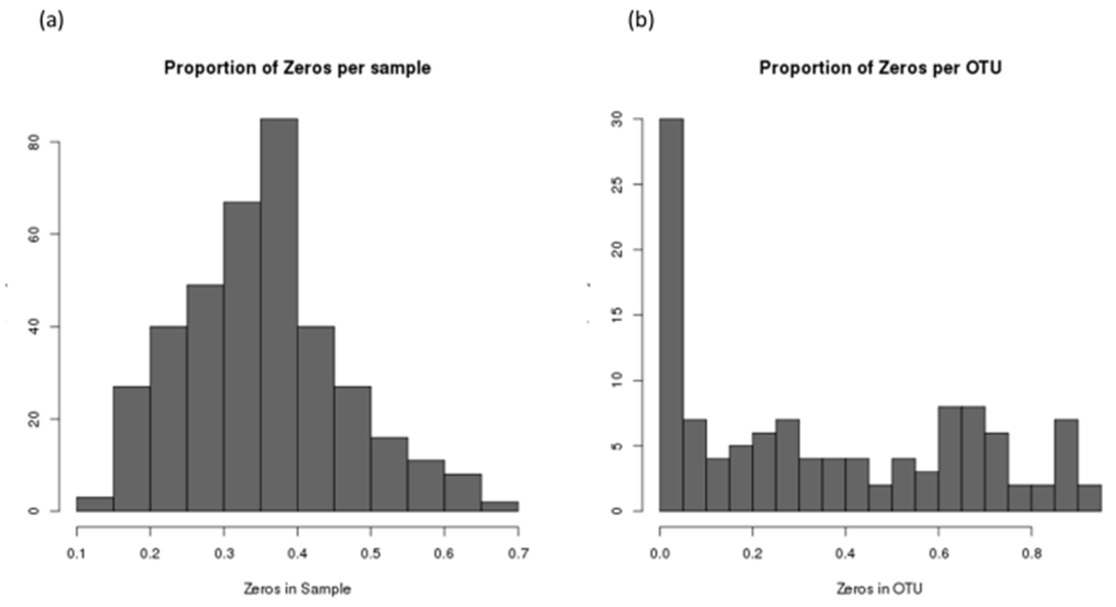

Figure S1. Proportion of zeroes per sample (figure S1a) and per OTU (figure S1b).

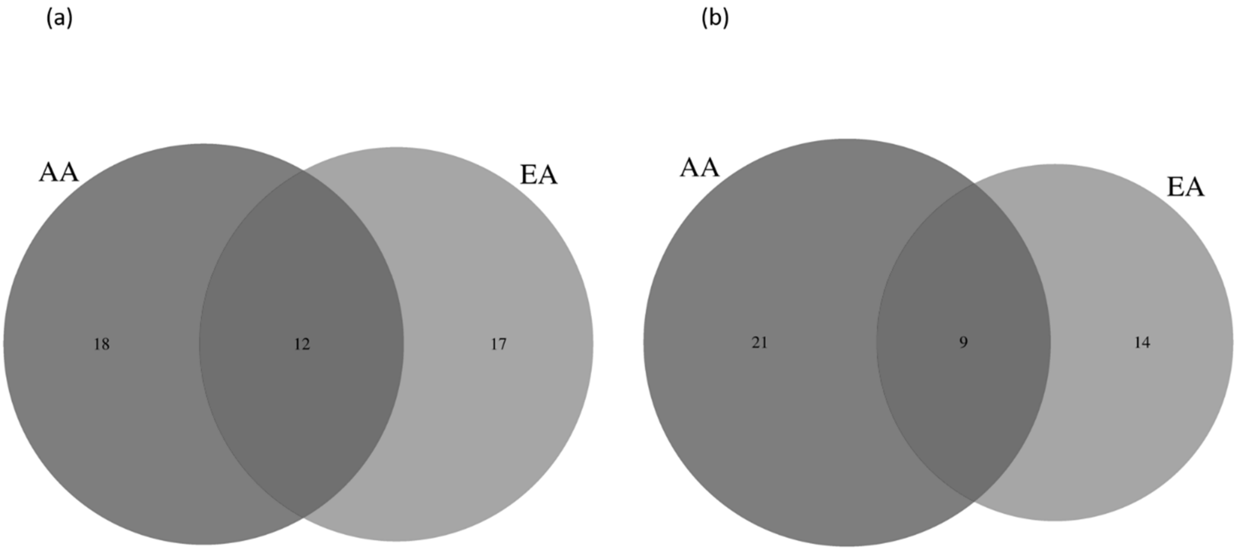

Figure S2. Venn diagram representing numbers of genera statistically significantly associated with ENL at baseline (figure S2a) and after intervention (figure S2b) by race. A greater number of genera overlap between AA and EA at baseline compared to postintervention supporting a differential effect of the intervention on the microbiome that varied by race.
